# Psoriasiform skin disease in transgenic pigs with high-copy ectopic expression of human integrins α2 and β1

**DOI:** 10.1242/dmm.028662

**Published:** 2017-07-01

**Authors:** Nicklas Heine Staunstrup, Karin Stenderup, Sidsel Mortensen, Maria Nascimento Primo, Cecilia Rosada, Torben Steiniche, Ying Liu, Rong Li, Mette Schmidt, Stig Purup, Frederik Dagnæs-Hansen, Lisbeth Dahl Schrøder, Lars Svensson, Thomas Kongstad Petersen, Henrik Callesen, Lars Bolund, Jacob Giehm Mikkelsen

**Affiliations:** 1 Department of Biomedicine, Aarhus University, 8000 Aarhus C, Denmark; 2 iPSYCH The Lundbeck Foundation Initiative For Integrative Psychiatric Research, Denmark; 3 iSEQ, Centre for integrative sequencing, Aarhus, Denmark; 4 Department of Dermatology, Aarhus University Hospital, 8000 Aarhus C, Denmark; 5 Department of Skin Inflammation Pharmacology, LEO Pharma, 2750 Ballerup, Denmark; 6 Department of Animal Science, Aarhus University, 8830 Tjele, Denmark; 7 Department of Veterinary Reproduction and Obstetrics, Faculty of Life Sciences, University of Copenhagen, 1870 Frederiksberg C, Denmark; 8 Department of NME Ideation, LEO Pharma, 2750 Ballerup, Denmark; 9 HuaDa JiYin (BGI), Shenzhen 518083, China

**Keywords:** DNA transposition, Integrin, Pig cloning, Psoriasis, Skin inflammation, Sleeping Beauty

## Abstract

Psoriasis is a complex human-specific disease characterized by perturbed keratinocyte proliferation and a pro-inflammatory environment in the skin. Porcine skin architecture and immunity are very similar to that in humans, rendering the pig a suitable animal model for studying the biology and treatment of psoriasis. Expression of integrins, which is normally confined to the basal layer of the epidermis, is maintained in suprabasal keratinocytes in psoriatic skin, modulating proliferation and differentiation as well as leukocyte infiltration. Here, we generated minipigs co-expressing integrins α2 and β1 in suprabasal epidermal layers. Integrin-transgenic minipigs born into the project displayed skin phenotypes that correlated with the number of inserted transgenes. Molecular analyses were in good concordance with histological observations of psoriatic hallmarks, including hypogranulosis and T-lymphocyte infiltration. These findings mark the first creation of minipigs with a psoriasiform phenotype resembling human psoriasis and demonstrate that integrin signaling plays a key role in psoriasis pathology.

## Abstract (precis)

**Summary:** A cloned porcine disease model to advance topical treatment in the debilitating skin disorder psoriasis.

## INTRODUCTION

Psoriasis vulgaris is an inflammatory and chronic cutaneous disorder affecting an estimated 2-3% of the Caucasian population worldwide. Main clinical hallmarks include well-demarcated thick, red and scaly skin plaques, whereas key histological hallmarks include (i) angiogenesis and dilation of blood vessels causing macroscopically visible erythema; (ii) dermal and epidermal infiltration of activated leukocytes (T-lymphocytes, dendritic cells, macrophages, and neutrophils); (iii) hyperproliferation of keratinocytes leading to keratinocyte hyperplasia (acanthosis) and the formation of rete ridges; (iv) retention of nuclei in corneocytes (parakeratosis) related to the atypical keratinocyte proliferation and differentiation; and (v) reduction or absence of the granular layer (hypogranulosis) (Nograles et al., 2010; Bowcock and Krueger, 2005; Lowes et al., 2007; Svensson et al., 2012). The lack of naturally occurring psoriasis in laboratory animals has encouraged the generation of genetically modified animal models with phenotypes reminiscent of psoriasis. Such models have helped demonstrate that vascular changes, epidermal hyperplasia and cell-mediated immunity are inter-related prerequisites for disease development (Schon, 2008; Wagner et al., 2010; Gudjonsson et al., 2007), but no model has been able to capture all histological and clinical traits of psoriasis. The immense concordance between human and porcine skin architecture makes pig models for skin disorders highly attractive for the study of disease development and treatment.

Psoriasis is a multifactorial disorder and a variety of genetically and environmental factors are involved in the etiology of the disease. However, it is evident that both dysregulation of the immune system as well as keratinocyte hyperproliferation are prerequisites for disease development. Hence, psoriasis has been associated with increased levels of inflammatory markers including interleukin (IL)-23, IL-17A and nuclear factor (NF)-κB, but also with markers of keratinocyte homeostasis such as signal transducer and activator of transcription 3 (STAT3) and keratinocyte growth factor (KGF) (Sano et al., 2005; Finch et al., 1997; McKenzie and Sabin, 2003; Nair et al., 2008).

In the epidermis, proliferation is normally confined to the stratum basale, where keratinocyte stem cells are attached to the basal membrane through integrins (Hotchin et al., 1995). Transit-amplifying cells leave the stratum basale and undergo a limited number of cell divisions before they commit to spatiotemporal differentiation as they ascend. Acting as a negative regulator of differentiation, integrin expression is lost in the process (Brakebusch et al., 2000). Hence, cells strongly positive for β1 integrin are indicative of stem cells, whereas cells expressing low levels of β1 integrin are transient-amplifying cells, and β1 integrin-negative cells are post-mitotic, differentiating cells. Notably, transgenic suprabasal expression of integrins promotes oncogenesis, emphasizing their pro-proliferative function (Carroll et al., 1995).

Integrins constitute a superfamily of glycosylated, heterodimeric transmembrane receptors composed of an α- and a β-subunit, exercising functions in cell adhesion as well as inside-out and outside-in signaling. The presence of active α2β1 integrin is normally confined to the stem cell-containing stratum basale, where it binds collagen as part of focal adhesions. In wound healing and psoriatic lesions, however, expression continues into the suprabasal layers. On this basis, transgenic mice have been generated that express integrin subunits driven by the involucrin promoter, which is specifically active in suprabasal keratinocytes. Induced skin irritation of such α2β1 transgenic mice led to a chronic condition resembling psoriasis with persistent hyperplasia, cutaneous influx of leukocytes and development of microabscesses, as well as secretion of pro-inflammatory cytokines (Carroll et al., 1995; Haase et al., 2001; Teige et al., 2010).

Suprabasal presence of the β1 integrin subunit has been shown to induce phosphorylation of the epidermal growth factor receptor (EGFR), even in the absence of EGFR ligands, which leads to activation of the cell cycle-stimulating MAPK/ERK pathway (Yu et al., 2000; Moro et al., 1998). Furthermore, the engineered suprabasal expression of β1 integrin triggers release of the potent pro-inflammatory cytokine interleukin 1α (IL-1α) in a ligation-independent manner, which further stimulates the MAPK/ERK pathway (Haase et al., 2001; Hobbs and Watt, 2003).

We have previously described the generation of transgenic pigs featuring ectopic expression of either human integrin α2 or integrin β1 (Staunstrup et al., 2012). These animals presented with an aberrant inflammatory profile in the skin but no visually apparent skin abnormality. Here, we describe the generation and evaluation of double-transgenic Göttingen minipigs (*Sus scrofa domestica*) featuring suprabasal expression of both human α2 and β1 integrin. Our findings document the development of a severe skin phenotype with evidence of psoriasiform epidermal hyperplasia and inflammation in minipigs engineered with multiple copies of the expression cassette encoding both α2 and β1 integrins.

## RESULTS

### Production of double-transgenic pigs expressing human *ITGA2* and *ITGB1*

**Fig. 1.**
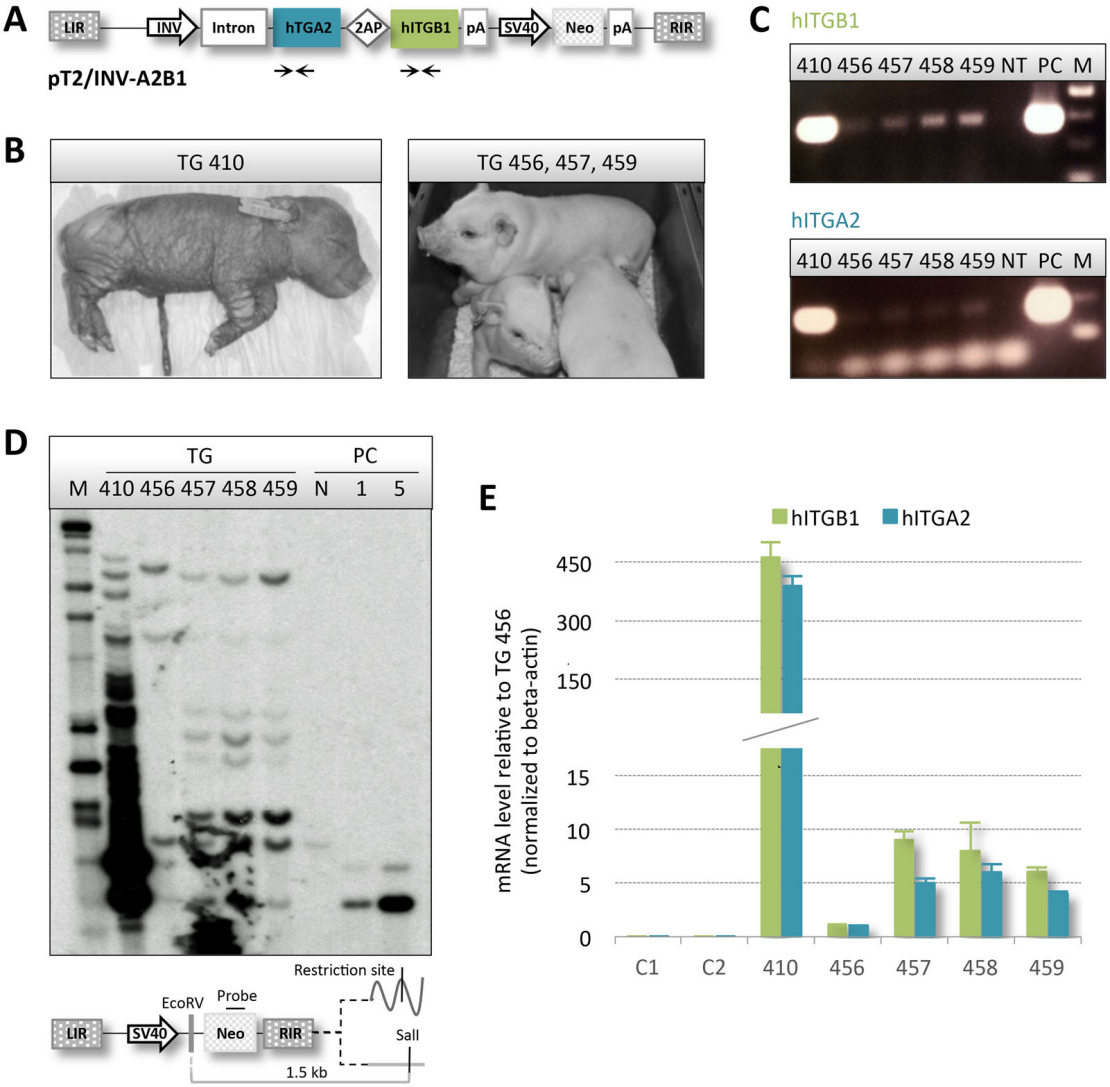
**Multi-copy transgenic animals verified by genotyping and expression analysis.** (A) Schematic illustration of the SB transposon vector holding the bicistronic integrin expression cassette controlled by the skin-specific INV promoter. Primers used for detection of the human *ITGA2* and *ITGB1* genes are indicated. LIR and RIR, SB transposon left and right inverted repeats; CMV, SV40 and INV, cytomegalovirus, simian virus 40 and human involucrin promoter; Intron, first intron of the involucrin gene; 2AP, viral 2A-like peptide; hITGA2 and hITGB1, human integrin α2 and β1; pA, polyadenylation sites; Neo, neomycin resistance gene. (B) Perinatally deceased pig TG-410 presenting with cutaneous abnormalities as well as surviving pigs TG-456, TG-457 and TG-459. (C) Semi-quantitative genomic PCR amplification of segments of *ITGA2* and *ITGB1* genes. Equal amounts of gDNA extracted from cultured primary fibroblasts derived from the five transgenic animals plus a non-transgenic control was utilized in the reaction and run on an agarose gel. NT, non-transgenic control; PC, plasmid control; M, 100 bp marker. (D) Genotyping by Southern blot with *Eco*RV/*Sal*I-digested gDNA from the five cloned pigs. Schematic illustration of the restriction products, as well as the binding site of the neomycin-specific probe. Genomic DNA from a non-transgenic pig was spiked with 0, 1 or 5 copies of pT2/INV-A2B1 and applied as a control. The number of insertions ranges from 5 to >50 in the five cloned animals, confirming a transgenic status. Two identical bands appear in all lanes and are presumably non-specific. (E) RT-qPCR on total RNA from two non-transgenic controls C1 and C2 and the five transgenic animals. Presence of the heterologous transcripts was demonstrated in all transgenic animals in a copy-number-dependent manner. Figures are normalized to endogenous β-actin and the data are presented as mean values±s.d. of biological triplicates.

To co-express human integrins α2 and β1, we produced Sleeping Beauty (SB) DNA transposon-based vectors carrying human *ITGA2* and *ITGB1* genes in a bicistronic expression cassette. Using a CMV promoter to drive expression, production and correct translational processing of α2 and β1 integrin from genomically integrated transposons was verified in mouse fibroblasts (Fig. S1). To generate transgenic pigs with ectopic expression of the two integrins, we exchanged the promoter with the involucrin (INV) promoter, resulting in the plasmid pT2/INV-A2B1 carrying the 12.3 kb SB vector encoding both α2 and β1 integrin (Fig. 1A). Expression from the SB vector was verified in stably transfected mouse fibroblasts (Fig. S2). Donor cells for hand-made cloning (HMC) were produced by insertion of the T2/INV-A2B1 transposon in Göttingen primary fibroblasts by co-transfection with pCMV-SB100X encoding the hyperactive SB100X transposase (Mátés et al., 2009). Approximately 20 G418-resistant colonies were collected and pooled and subsequently utilized as donor cells in the HMC procedure. The blastocyst rate was 36%, and a total of 70 reconstituted embryos were surgically transferred to a single recipient sow, which gave rise to a total of 5 piglets (Table S1, Fig. S3). One piglet was stillborn (TG-458) and one with severe skin abnormalities (TG-410, Fig. 1B) died shortly after delivery. The remaining piglets (TG-456, TG-457 and TG-459; Fig. 1B) presented no gross abnormalities, were housed together and gained weight according to the growth curve of the herd.

Genomic DNA extracted from ear biopsies derived from all cloned animals was used for insert detection by PCR and Southern blot analysis using a *neo* probe. Specific PCR amplification of fragments of the human *ITGB1* and *ITGA2* genes indicated that the transgenes were indeed integrated in the genomes of all cloned animals and, notably, with higher copy numbers in pig TG-410 resulting in massive PCR amplification (Fig. 1C). An estimation of the number of transposon copies in each piglet was achieved by Southern blot analysis (Fig. 1D, Fig. S4). Interestingly, TG-410 contained multiple transposon inserts, presumably over 50 copies. TG-456 harbored only 5 copies, of which one most likely originated from random insertion of the plasmid. Intriguingly, the remaining three piglets displayed identical band patterns with an estimated 8 copies, of which a single insert appeared to be a transposase-independent insertion, suggesting that these three animals arose from the same donor cell clone.

Keratinocytes were expanded *in vitro* from skin explants for at least 3 weeks, allowing keratinocytes to differentiate and, thus, to switch on the INV promoter. Total RNA was extracted from pooled outgrowths from each of the five transgenic pigs and two age-matched wild-type controls (WT C1 and C2) and subsequently used for RT-qPCR (Fig. 1E). A cycle threshold (*C*t) value above background could not be detected in the non-transgenic animals, whereas a marked signal of integrin-encoding mRNA, using both *ITGA2* and *ITGB1* primer sets, was recorded in all transgenic animals, indicating that the complete polycistronic transcript was being produced. Notably, we observed an apparent coherence between copy numbers and expression levels. Hence, pig TG-456 with the fewest insertions also had the lowest level of transcripts, whereas TG-410 exhibited levels of RNA at least 400-fold higher than TG-456. The three genetically identical animals displayed comparable levels of expression, but at a much lower level than TG-410. In summary, these findings confirmed that all five cloned animals were transgenic and that one multi-copy transgenic pig showed a severe skin phenotype.

### Membrane localization of human α2 and β1 integrin in keratinocytes derived from transgenic pigs

**Fig. 2.**
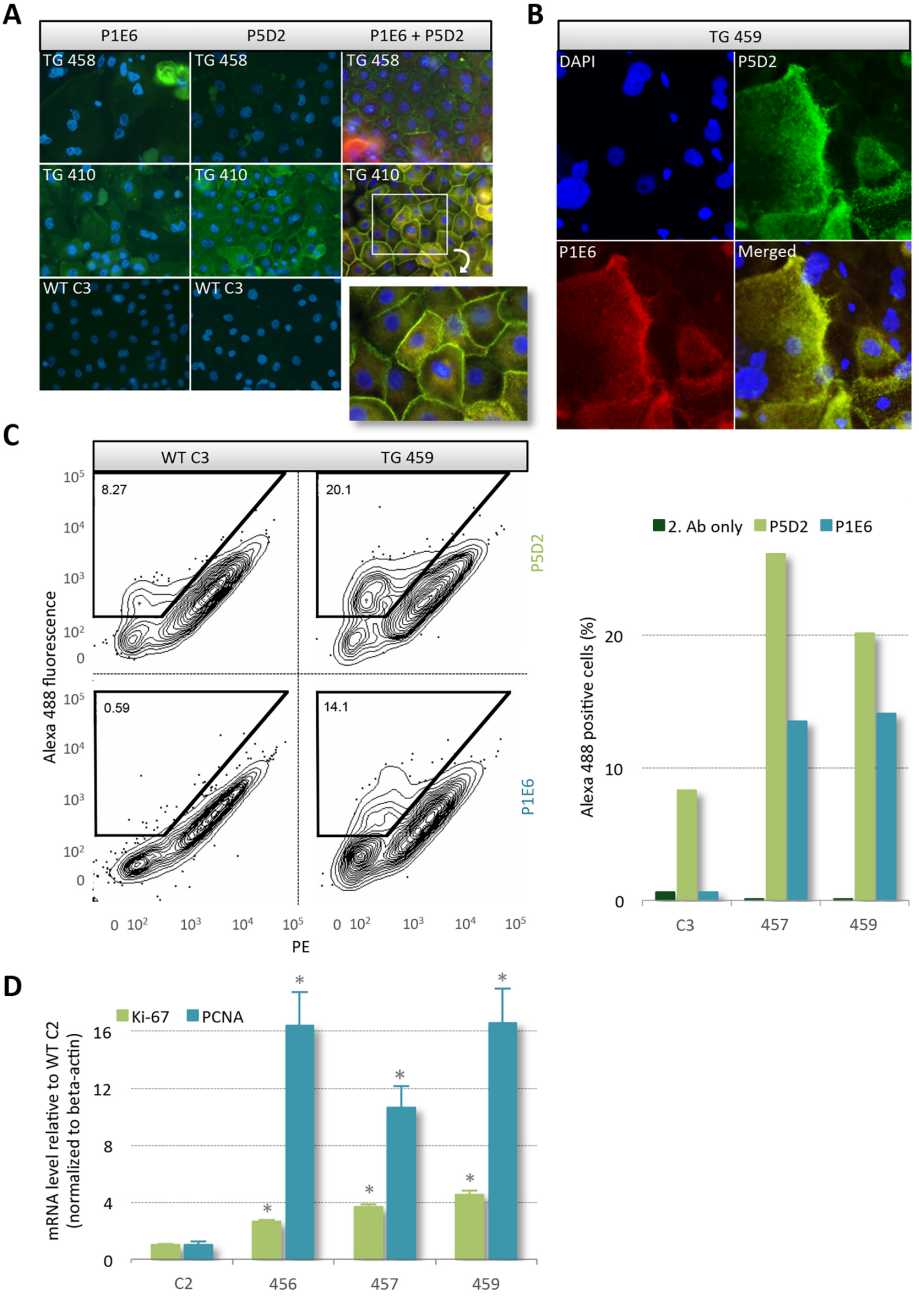
**Translation and correct distribution of heterologous α2 and β1 integrin.** (A) Representative immunocytochemistry images using human-specific antibodies against α2 (P1E6) and β1 (P5D2) integrin performed on permeabilized cultured primary keratinocytes from two transgenic pigs (TG-410 and TG-458) and a non-transgenic control (WT-C3). The cells were either stained singly or in combination. DAPI was used to stain the nucleus. Detection of integrin was restricted to transgenic cells. (B) Representative images from confocal fluorescence microscopy of saponin permeabilized keratinocytes from pig TG-459 stained with the nucleus marker DAPI along with anti-human antibodies against α2 (P1E6) and β1 (P5D2) integrin. Integrin accumulates at the plasma membrane and especially at cell-cell adhesion sites, suggesting correct processing of the peptide. (C) Flow cytometry analysis of unpermeabilized keratinocytes from transgenic pigs, TG-457 and TG-459, and control pig, WT C3, stained with α2 (P1E6) or β1 (P5D2) integrin-specific antibodies. Staining solely with the secondary antibody served as a control. Representative dot plots comparing fluorescence from pig WT-C3 and TG-459 are depicted. (D) Quantitative RT-PCR on total RNA extracted from skin biopsies obtained from transgenic pigs TG-456, TG-457 and TG-459, and the non-transgenic age-matched control WT-C2. Relative mRNA levels of *Ki-67* and *PCNA* are depicted normalized to endogenous *β-actin*. Data presented as mean values±s.d. of biological triplicates where the difference is calculated using the Student's *t*-test.

Only heterodimeric integrin complexes can accomplish the canonical function of integrins as transmembrane receptors. To track membrane localization of heterologous human α2 and β1 proteins, primary keratinocytes from the transgenic pigs TG-410 and TG-458, as well as from the non-transgenic control WT-C3 were expanded *in vitro* and then immunostained using primary antibodies against human α2 (P1E6) and β1 (P5D2) integrin and a green fluorophore-conjugated secondary antibody (Fig. 2A). The presence of both exogenous integrins was evident in keratinocytes from both transgenic animals. Furthermore, staining was not observed in P1E6- or P5D2-stained keratinocytes derived from WT-C3. Notably, the more intense staining in TG410-derived keratinocytes compared with cells from TG-458 correlated with the higher mRNA level seen in Fig. 1E. A merged picture of sequentially stained cells derived from the transgenic animals employing a green and red fluorophore-conjugated secondary antibody demonstrated co-localization of the two integrins at the cell membrane (Fig. 2A, right column). Furthermore, it was evident that the fluorescence intensity increased in ascending cells, which is in concordance with the spatiotemporal activity of the INV promoter being limited to differentiation-committed cells. Confocal microscopy of dual-stained keratinocytes derived from TG-459 further supported the notion of membrane anchorage of the ectopically expressed integrins (Fig. 2B). A nearly complete overlap in the localization of α2 and β1 integrin was visible in the cultured keratinocytes. In most cells, transport vesicles that stained positive for both α2 and β1 integrin subunits were visible, further emphasizing a correct cellular function and distribution of exogenous integrin complexes (Fig. S5). Of note, there was no bleed-through of the fluorescent signals.

A third evaluation of the human α2 and β1 integrin distribution was made by flow cytometry. A subpopulation of the cultured porcine keratinocytes were harvested and stained with P5D2 or P1E6 without permeabilizing the cell membrane. Given that both antibodies recognize an extracellular epitope, this ensured that only membrane-protruding integrin subunits were marked. The percentage of α2 and β1 integrin-presenting cells in the heterogeneous pool of keratinocytes, all at variable stages of differentiation, was significantly higher in cells originating from the transgenic animals compared with cells from the control pig (WT C3), although the β1 antibody showed some cross-specificity (Fig. 2C). In accordance, quantitative PCR on cDNA revealed a significant increase in the relative level of the pro-proliferative markers antigen Ki-67 (*Ki-67*) and proliferation cell nuclear antigen (*PCNA*) in the three transgenic pigs compared with the control (*P*<0.0001) (Fig. 2D). This is in line with previous findings showing that suprabasal epidermal expression of human integrins in mice leads to acanthosis (Carroll et al., 1995) and suggested that homing of transgenic integrins to the membrane was active. In summary, these data support the notion that exogenous integrins were correctly processed and displayed at the cell membrane, allowing interaction with extracellular ligands and endogenously expressed membrane receptors.

### Generation of piglets with severe acanthosis by re-cloning of TG-410

**Fig. 3.**
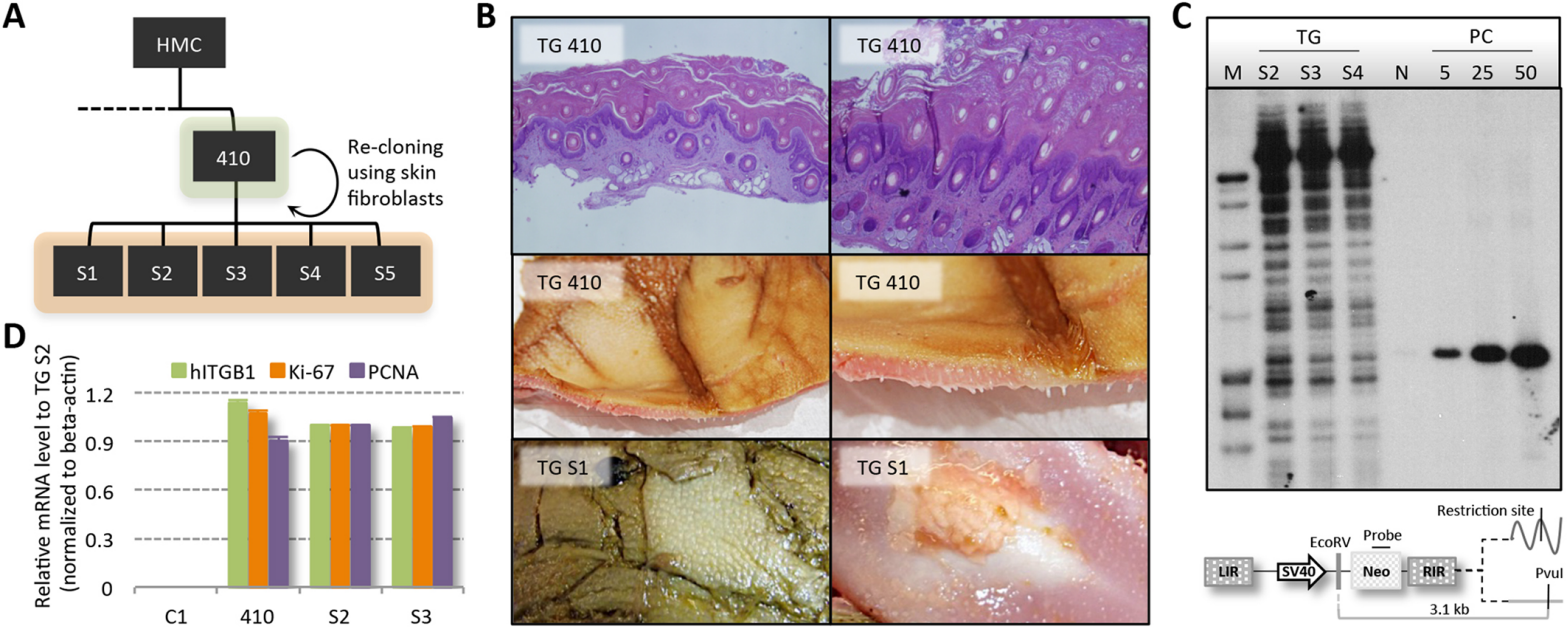
**Re-cloning of TG-410 produced five minipigs with a parental-type phenotype.** (A) Epithelial fibroblasts from TG-410 were used for re-cloning resulting in the delivery of TG S1-S5. (B) H&E staining of TG-410 skin section (top row). Photographs of the skin of TG-410 and TG-S1, indicating deregulated keratinocyte proliferation (middle and bottom row). (C) Genotyping by Southern blot using *Eco*RV/*Pvu*I-digested gDNA from three (TG-S2, TG-S3 and TG-S4) re-cloned pigs. Genomic DNA from a non-transgenic pig was spiked with 5, 25 or 50 copies of pT2/INV-A2B1 and applied as a control. The pattern and number of bands visible is identical among the three re-cloned animals and confirming the transgenic status and clonality. (D) Quantitative RT-PCR on total RNA extracted from skin biopsies taken from transgenic pigs TG-410, TG-S2 and TG-S3, and the non-transgenic age-matched control WT-C1. Relative mRNA levels of human *ITGB1*, *Ki-67* and *PCNA* are depicted normalized to endogenous β-actin. Data presented as mean values±s.d. of biological triplicates.

Owing to the lack of visual phenotypic changes in the skin of low-copy transgenic pigs, we investigated whether chronic skin inflammation could be induced in pigs TG-456 and TG-459 using potent chemical inducers of inflammation. Although epidermal thickness and the transcription level of inflammation markers were increased by the treatment, they reverted to near-baseline levels post treatment (Figs S6,S7). Blood profiling revealed increased numbers of eosinophils and neutrophils and decreased numbers of lymphocytes in the transgenic pigs (Table S1). Interestingly, a moderately higher neutrophil load in peripheral blood is consistently found in psoriatic patients (Zanolli et al., 1989). Also, the lower number of lymphocytes is in agreement with findings in moderate-to-severe psoriasis patients and in α2/β1 transgenic mice (Teige et al., 2010; Lowe et al., 1981; Xia et al., 2003). Together, these findings suggested that low-copy number transgenic pigs were indeed in a state of inflammatory acuteness but at a level inadequate to surpass disease threshold. We therefore chose to focus on pig TG-410, which according to the autopsy had slightly immature but normally positioned internal organs. The lungs of TG-410 were atelectatic in about 70% of the tissue, and agenesis of the gall bladder was evident. The skin was discolored by meconium and showed signs of hyperkeratosis and acanthosis. Based on these findings, we decided to re-clone TG-410. Skin fibroblasts isolated from an ear clip of TG-410 were propagated and used for HMC. Achieving a blastocyst rate of 24%, a total of 92 reconstituted embryos were surgically transferred to a single recipient sow, resulting in five piglets (TG-S1 to TG-S5) delivered at term (Fig. 3A, Table S2, Fig. S3). Unfortunately, the five pigs were either stillborn or perished shortly after birth. They all presented the same phenotypic traits as TG-410 with whole-body thickening of the skin, even on the tongue, primarily attributed to an extensive stratum corneum layer (Fig. 3B). As with TG-410, autopsy of TG-S1 and TG-S2 revealed that the internal organs were normally developed and positioned, but also that the lungs were partially atelectatic. For one of the pigs, TG-S1, serohemorrhagic fluid could be observed in the abdominal and thoracic cavity.

Southern blot analysis performed on skin-derived DNA from the re-cloned pigs, confirmed kinship and transgenic status with more than 50 stably integrated copies of the transgene cassette (Fig. 3C). Transcriptional activity of the transgene cassette in the re-cloned animals and downstream hyperproliferative effects were evaluated by RT-PCR using primer sets for human *ITGB1*, *Ki-67* and *PCNA* on primary keratinocytes isolated from TG-S1, TG-S2, TG-410 and the control WT-C1 (Fig. 3D). Notably, all three transgenic pigs showed levels of expression that were highly elevated relative to the control animal. Also, no marked differences in expression of any of the three genes were observed among the three transgenic pigs. These findings confirmed that the re-cloned animals were genotypically and phenotypically identical to TG-410 and, thus, that ectopic co-expression of human integrins induced severe skin abnormalities in these animals.

### Histological changes in the re-cloned transgenic pigs with psoriasiform characteristics

**Fig. 4.**
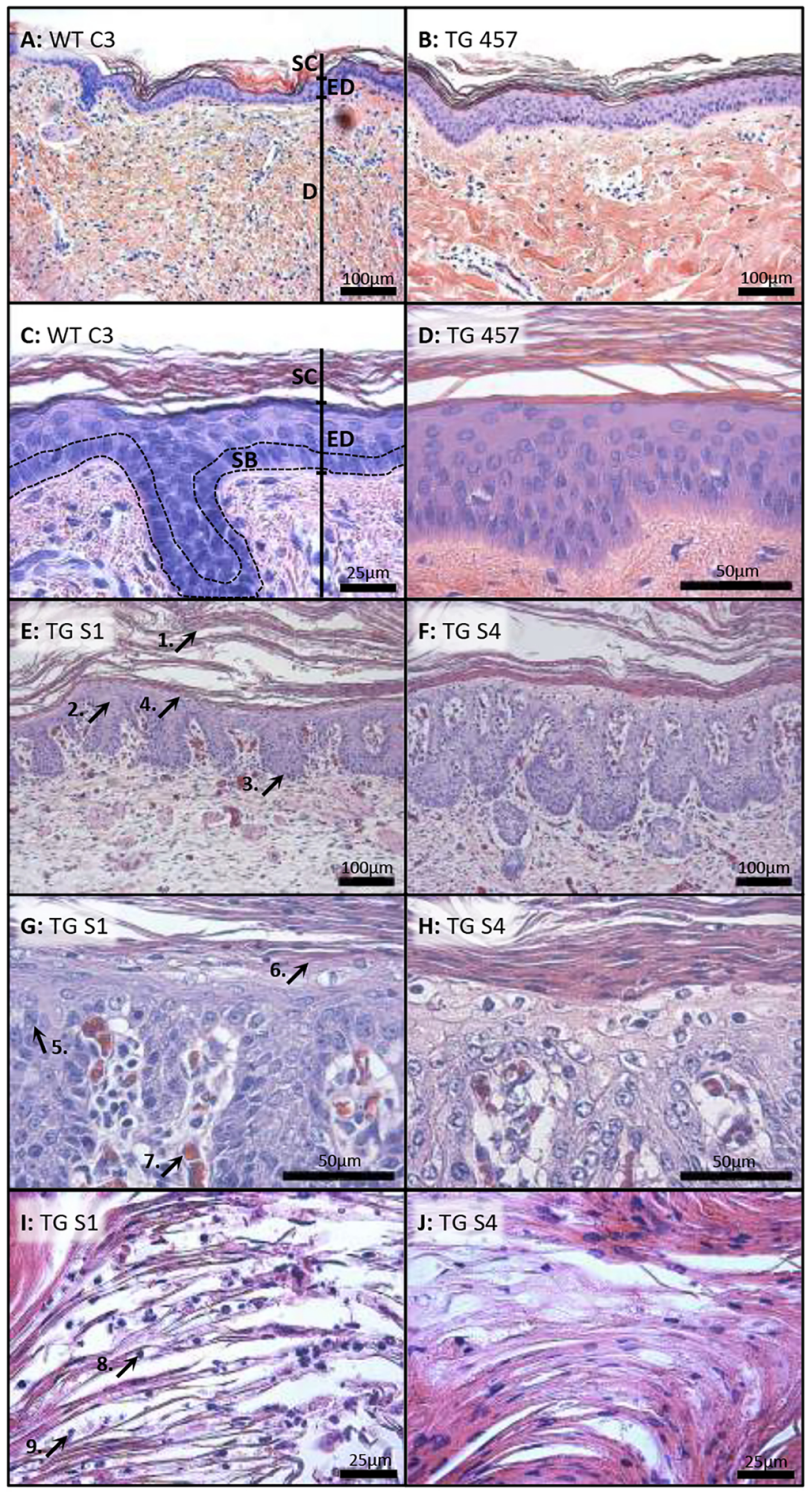
**Skin from multi-copy transgenic pigs exhibits parakeratosis, leukocyte infiltration and hyperkeratosis.** (A-J) H&E staining of cross-sectioned skin biopsies taken from the non-transgenic pig WT-C3 (A,C), the original transgenic animal TG-457 (B,D) and the re-cloned transgenic pigs TG-S1 and TG-S4 (E-J). The skin of multi-copy transgenic pigs shows hyperkeratosis (arrow 1), acanthosis (arrow 2), formation of rete ridges (arrow 3), but the absence of papillomatosis (arrow 4). In addition, there are suprabasal mitotic cells (arrow 5), clear hypogranulosis (arrow 6) and vascular dilation (arrow 7). The stratum corneum contains nucleated keratinocytes, parakeratosis (arrow 8) as well as granulocytes (arrow 9). SC, stratum corneum; ED epidermis; D, dermis. Dashed lines surround the stratum basale.

To investigate the skin pathology in the multi-copy transgenic pigs, paraffin sections of skin biopsies from six transgenic pigs (TG-457, TG-S1 to TG-S5) and three non-transgenic controls (WT-C3, WT-C7 and WT-C8) (Fig. S3) were stained with Hematoxylin and Eosin (H&E; Fig. 4, Fig. S8). The overall architecture of the skin in TG-457 was intact when judged against WT-C3 (Fig. 4A-D), and signs of keratinocyte dysregulation or influx of inflammatory cells were not evident. By contrast, the skin architecture of the multi-copy transgenic animals was grossly altered (Fig. 4E,F, Fig. S8), and the skin of these animals was clearly thickened as a result of hyperkeratosis and acanthosis. The stratum corneum was several folds thicker in the transgenic animals, displaying a seemingly chaotic flaking. The epidermis was also denser in TG-S1 and TG-S4, and peculiarly, the stratum corneum contained hair follicle structures, possibly due to a rapid keratinocyte turnover (Fig. S9). Furthermore, rete ridges (downwards extending epidermal ‘fingers’) were identifiable, often fused to adjacent ridges, whereas no signs of papillomatosis (papillary projections of the epidermis forming an undulating surface) were evident. Magnification of the epidermis (Fig. 4G,H, Fig. S8) revealed a high number of mitotic cells in the suprabasal layer (stratum spinosum), supporting the notion of a hyperproliferative state, presumably instigated by the high expression of the heterologous integrins. Interestingly, the stratum granulosum was largely absent (hypogranulosis) in the transgenic pigs, which could be ascribed to the inhibited terminal differentiation of the keratinocytes as a classical hallmark of psoriatic skin. Equally notable was the dilation of vessels in the papillary dermis and the presence of granulocytes, mostly in the form of neutrophils. Multiple non-terminally differentiated nucleated cells were also seen in the stratum corneum (Fig. 4I,J, Fig. S8), which revealed clear signs of parakeratosis.

### Infiltration of immune cells in the skin of multi-copy transgenic pigs

**Fig. 5.**
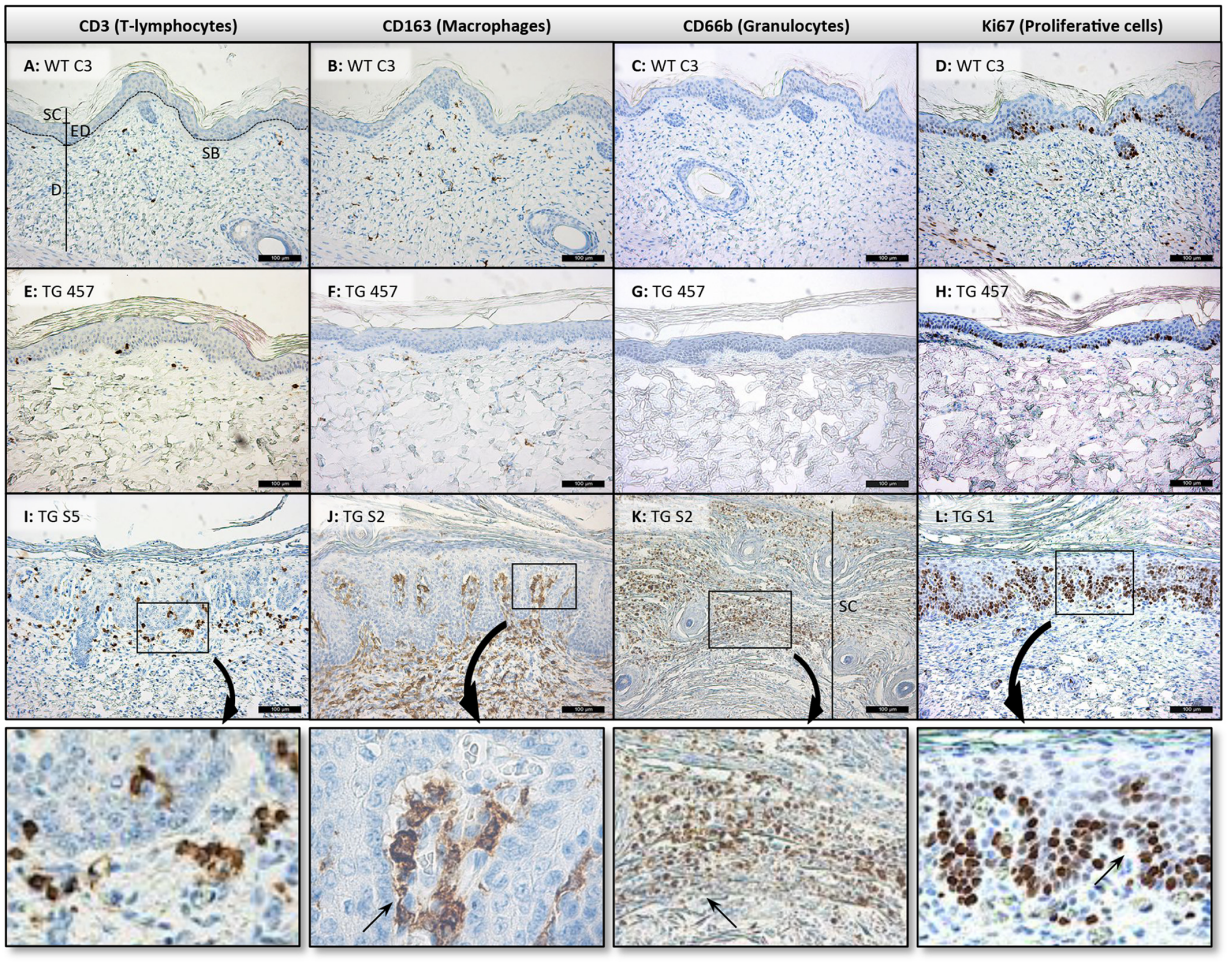
**Immunohistopathology reveals cutaneous infiltration of T-lymphocytes, macrophages and granulocytes, in addition to epidermal hyperproliferation.** (A-L) Representative immunostaining images of FFPE skin sections from transgenic (TG-457, TG-S1, TG-S2 and TG-S5; E-L) and control (WT-C3; A-D) animals. T-lymphocytes (CD3), macrophages (CD163), granulocytes (CD66b) and proliferation (Ki67) markers were used. Boxed regions in I-L are shown enlarged below. SB, stratum basale; SC, stratum corneum; ED epidermis; D, dermis. Scale bars: 100 μm.

To analyze cutaneous influx of immune cells, skin sections from six transgenic pigs (TG-457 and TG-S1 to TG-S5) plus three non-transgenic age-matched controls (WT-C3, WT-C7 and WT-C8) were stained for cellular markers for T-lymphocytes (CD3), macrophages (CD163) and granulocytes (CD66b), as well as for proliferation (Ki-67) (Fig. 5, Fig. S10). Whereas only a few scattered CD3^+^ T-lymphocytes were visible in the controls (Fig. 5A), a slightly increased number was evident in the skin of TG-457 (Fig. 5E). In the skin of multi-copy transgenic animals, however, we observed a markedly increased influx of T-lymphocytes along the papillary epidermis and throughout the suprabasal layer (Fig. 5I, Fig. S10). Moreover, staining for macrophages showed a massive infiltrate mostly confined to the dermal layer in all multi-copy transgenic pigs (Fig. 5J, Fig. S10), whereas the control animals and TG-457 showed a baseline presence of macrophages (Fig. 5B,F, Fig. S10). Staining for granulocytes (CD66b) was negative in wild-type animals, as well as in TG-457 (Fig. 5C,G, Fig. S10). However, aggregates of neutrophils were easily detectable in the stratum corneum of TG-S1 to TG-S5 (Fig. 5K, Fig. S10), providing evidence for the chemotactic recruitment of neutrophils characteristic of an ‘acute’ inflammatory state of psoriasis. We also stained skin sections for Ki67 and found scattered proliferating keratinocytes in the stratum basale of the control pigs and TG-457, with only a limited number of transient-amplifying cells present (Fig. 5D,H, Fig. S10). In contrast, basically all cells in the entire stratum basale in sections derived from all five multi-copy transgenic pigs were actively proliferating (Fig. 5L, Fig. S10). Moreover, large sections of the suprabasal layer were Ki67 positive (Fig. 5L), stressing the impact of integrin expression as a stimulator of cell division and a negative regulator of differentiation.

### Psoriasiform skin disease, and not dermatitis, in multi-copy ITGB1/ITGA2 transgenic minipigs

**Fig. 6.**
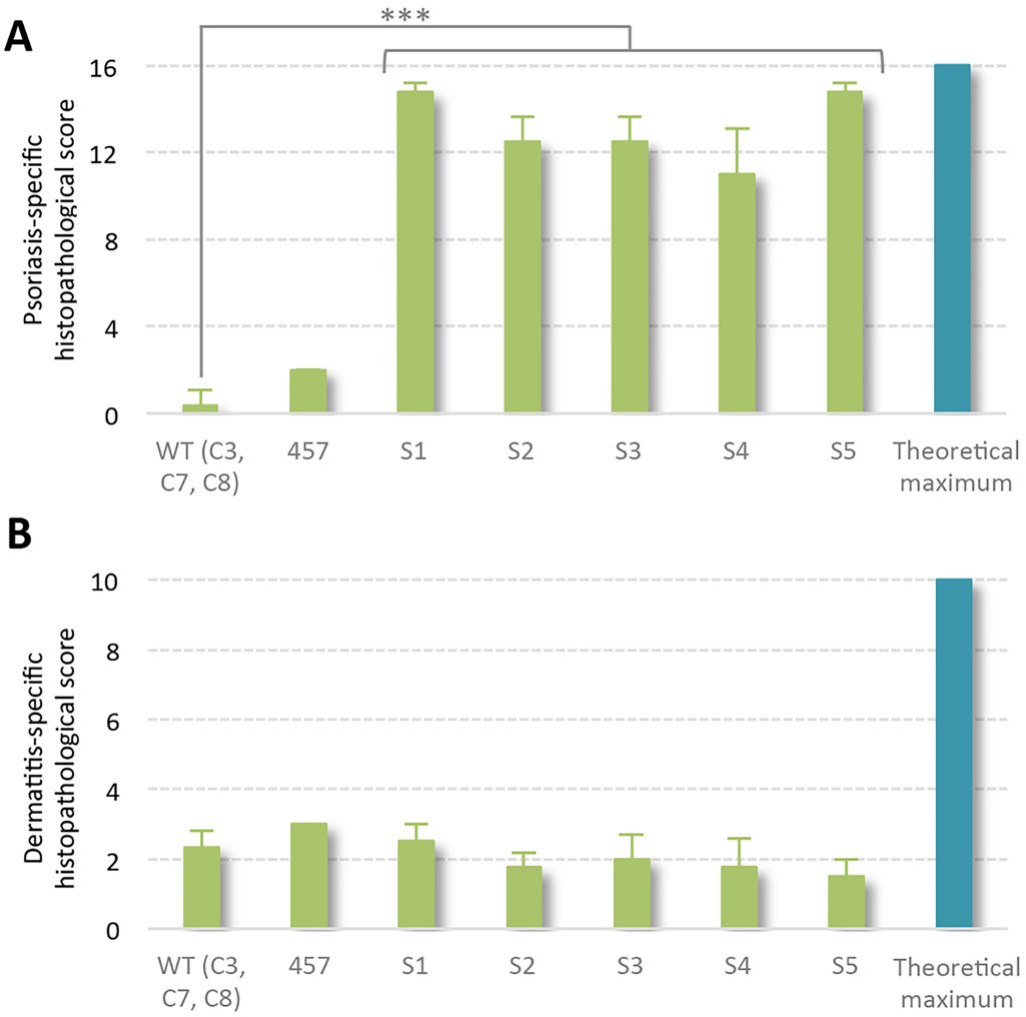
**Psoriasis- and dermatitis-specific histological scores point to psoriasis rather than dermatitis.** (A) Accumulated score for psoriasis distinct characteristics evaluating 2-4 skin sections of each transgenic animal and a combined WT group (WT-C3, WT-C7, and WT-C8) compared with the theoretical maximum. (B) Dermatitis-specific traits were assessed in 2-4 skin sections of each transgenic animal and a combined WT group (WT-C3, WT-C7 and WT-C8) along with the theoretical maximum. ***P<0.001, Fisher's exact test.

Based on Hematoxylin and Eosin-stained and immunostained tissue sections, a cumulative score for characteristic traits of psoriasis and dermatitis was given to three control pigs (WT-C3, WT-C7 and WT-C8) as well as six transgenic pigs (TG-457 and TG-S1 to TG-S5). The sections were blinded, after which they were independently assessed and granted a score for each trait from 0 to 2 (0, absent; 1, present; 2, severe). Seven psoriasis-specific identifiers and four dermatitis-specific markers, as well as two markers common for the two diseases were assessed (Fig. S11). A modest non-significant difference in the psoriasis-specific scoring between the controls and TG-457 was attributed to the partial loss of the stratum granulosum and mild exocytosis in the latter (Fig. S11). Nevertheless, these findings indicate that the expression of the heterologous integrins and not genetic disruption by the transgenic cassette itself was responsible for the observed phenotype. In contrast, all transgenic pigs derived from TG-410 obtained a psoriasis score close to the theoretically highest score, reaching a mean of 13.2 (s.d.±1.7) (Fig. 6). Notably, the deficit to the highest score could be almost exclusively ascribed to the lack of perivascular-located neutrophils in the transgenic pigs (Fig. S11). For comparison, the total score in control pigs was 0.3 (s.d.±0.7). These findings showed that a very consistent psoriasiform phenotype was present in piglets carrying multiple copies of the transgene cassette. In accordance, none of the pigs showed signs of dermatitis, and significant dermatitis-specific dissimilarities between transgenic and control animals were not evident. Taken together, a combined list of the interrogated pathological traits characteristic of psoriasis revealed a compelling concordance between the observed changes in the skin of the multi-copy transgenic pigs and expected changes found in human psoriasis (Table 1). In summary, our findings demonstrate that transgenic minipigs expressing high levels of α2 and β1 integrin in the skin develop severe skin inflammation showing disease characteristics consistent with a severe psoriasiform condition both at the macroscopic and cellular level.

**Table 1.**
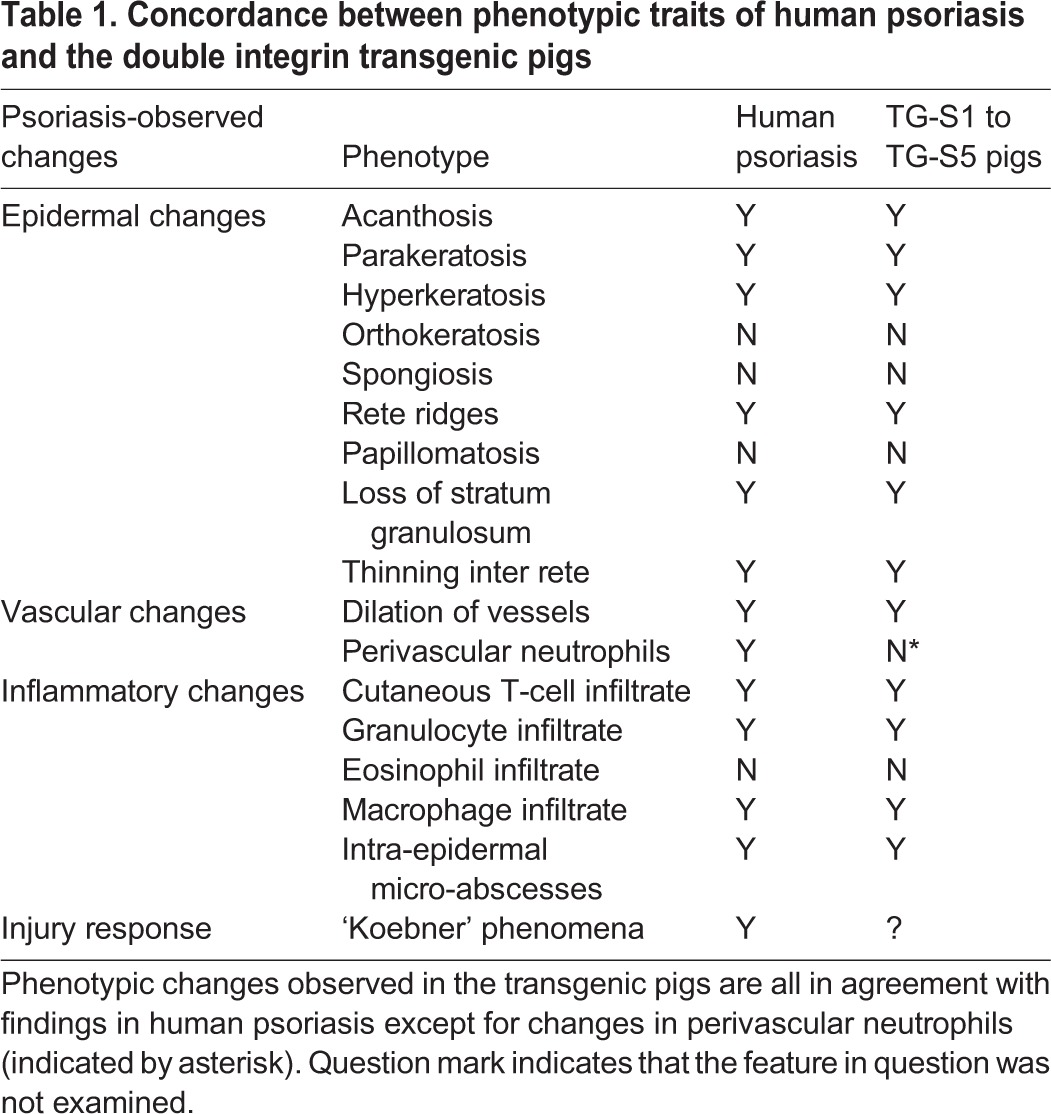
**Concordance between phenotypic traits of human psoriasis and the double integrin transgenic pigs**

## DISCUSSION

With two reported exceptions in monkeys, psoriasis has not been observed in animals other than humans (Zanolli et al., 1989; Lowe et al., 1981). Several transgenic and knockout murine models of psoriasis have been created (Sano et al., 2005; Xia et al., 2003; Groves et al., 1995; Bullard et al., 1996; Pasparakis et al., 2002), but common to all these models is that they fail to completely recapitulate all aspects of the disease in humans. Failure to model the disease in mouse skin may largely be due to crucial differences in skin morphology and immune response between humans and rodents, including keratinocyte turnover rate and differences in the subtypes of skin-homing T-lymphocytes (Berking et al., 2002; Khavari, 2006; Elbe et al., 1996; Ardavín, 2003). Xenotransplantation of human psoriatic skin onto SCID mice, therefore, still ranks as one of the most faithful models of the disease (Haftek et al., 1981; Kundu-Raychaudhuri et al., 2014).

The human-like skin architecture of pigs and the establishment of pig cloning from genetically modified somatic cells have made porcine models for human skin conditions attractive. However, besides the incidental finding of some psoriasiform characteristics in a transgenic pig with constitutive cutaneous expression of the hedgehog transcriptional activator, Gli2 (McCalla-Martin et al., 2010), porcine models of psoriasis-like conditions have not, to our knowledge, been described. We have previously described the generation of transgenic pigs with ectopic expression of either human integrin α2 or integrin β1 in the skin (Staunstrup et al., 2012). These minipigs displayed altered cytokine profiles in the skin, but did not develop a visible phenotype reminiscent of psoriasis. Here, we generate double-transgenic pigs featuring constitutive epidermal co-expression of integrin α2 and β1 and identify minipigs showing a severe skin inflammatory phenotype with a striking similarity to psoriasis.

Integrins are bidirectional-signaling molecules anchoring adherent cells to the extracellular matrix, but are also instrumental in regulating cell migration, survival and proliferation. Hence, ligated integrins in multi-component complexes at the cell membrane of adherent cells function as negative regulators of apoptosis and convey pro-proliferative signals. Under homeostasis, epidermal expression of integrins is confined to stem cell compartments of the stratum basale, suggesting that keratinocytes ascending through the suprabasal layer rapidly lose their proliferative potential and start to differentiate. Deregulation of epidermal homeostasis and chronic inflammation are characteristics of psoriasis. Notably, keratinocytes constitute a key node in this regard as they both belong to the innate immune system and are the primary cells of the epidermis. In line with this, faulty regulation of keratinocyte fate can lead to augmented release of leukocyte chemoattractants, such as IL-1α. Recruited active leukocytes release additional cytokines, possibly leading to a state of self-sustainable inflammation. We and others have previously shown that suprabasal expression of α2 and β1 integrins in transgenic animals leads to the augmented release of IL-1α (Haase et al., 2001; Staunstrup et al., 2012).

The first round of cloning produced four low copy-number transgenic pigs (TG-456 to TG-459) and a single piglet with >50 copies of the transgene cassette (TG-410), all featuring transgene transcription levels in skin correlating to the number of copies. Whereas the transgenic pigs harboring only 4-8 copies at first sight were indistinguishable from control animals, TG-410 presented with severe skin abnormalities. The correlation between transcription level and phenotypic appearance was a compelling indicator of heterologous integrin functionality. Whereas the nature of the high permissibility of the primary fibroblast that eventually led to TG-410 is unknown, only some of the insertions were mediated by SB transposition. As evaluated by Southern blotting, most of the inserted transgenes were organized in concatamers, probably through transposase-independent mechanisms (Chen et al., 2001; Dai et al., 2010). Expression and colocalization of membrane-homing human α2 and β1 integrins indicated correct processing of the peptides, including maturation of pre-β1 integrin and dimerization in the endoplasmic reticulum (Carroll et al., 1995; Koivisto et al., 1994). Along with the demonstrated expressional regulation of proliferation marker, this suggested that functional integrin dimers were indeed present in the cell membrane of keratinocytes in transgenic pigs.

Re-cloning of TG-410 resulted in piglets that were phenotypically and molecularly identical to TG-410 at birth, suggesting that the phenotypic traits directly reflected the genetic composition of the pigs. Intriguingly, histological assessment revealed traits consistent with psoriasis vulgaris. In all animals, hyperproliferation of basal and suprabasal keratinocytes was evident, leading to the formation of rete ridges, thinning of inter rete and acanthosis. It is important to note that the suprabasal integrin expression is not a corollary of inflammation as intradermal injection of cytokines into normal human skin has been shown not to induce such expression (Hertle et al., 1995). Previous studies have found that ectopic expression of integrins on suprabasal keratinocytes does not directly prompt cells in the stratum basale to enter mitosis (Romero et al., 1999). Rather, the augmented release of IL-1α from keratinocytes stimulates the MAPK/ERK and NF-κB pathways, leading to growth homeostasis and inflammatory responses (Murphy et al., 2000; Kaufman and Fuchs, 2000). We and others have previously reported a higher production of IL-1α from human *ITGB1* transgenic primary keratinocytes (Haase et al., 2001; Staunstrup et al., 2012).

The rapid turnover and faulty differentiation of ascending keratinocytes gave rise to parakeratosis, hyperkeratosis and hypogranulosis. An exacerbated release of cytokines and chemokines from the vast number of proliferating keratinocytes likely instigated a state of inflammation. Such an overstimulated release of lymphocyte-recruiting factors from human *ITGB1* transgenic pigs was shown earlier (Staunstrup et al., 2012). Interestingly, as one of several hallmarks of psoriatic skin (Lowes et al., 2007), dermal CD3^+^ cells clustered in the upper dermis of the transgenic animals. Overall, the release of recruitment factors ensures cutaneous influx of lymphocytes and macrophages, as well as stratum corneum-homing granulocytes forming Munro's microabscesses (Kim et al., 2014; Mitsui et al., 2012). Expansion of the microvascular system is another hallmark of psoriatic lesions that helps to nourish the hyperproliferating skin and permits massive extravasation of leukocytes. A marked dilation of tortuous dermal capillaries was palpable in the multi-copy transgenic pigs.

The only psoriasis-specific parameter absent in the skin of the transgenic pigs was the anticipated presence of perivascular neutrophils. However, as opposed to observations commonly found in dermatitis, no spongiosis (intercellular edema between keratinocytes) or orthokeratosis (hyperkeratosis without parakeratosis) could be observed in the skin of the transgenic pigs. Collectively, this argues in favor of a psoriasiform condition. Furthermore, the accumulation of cutaneous neutrophils and macrophages points to a self-perpetuating chronic/acute state of inflammation, as these cells potentiate both the T-cell response and keratinocyte deregulation. This, on the other hand, facilitates activation of the complement system by activated keratinocytes and a further release of pro-inflammatory molecules (Terui, 2000; Djemadji-Oudjiel et al., 1996; Willment et al., 2003).

Blinded evaluation of histological sections produced a cumulative psoriasis and dermatitis score for each of the transgenic pigs, as well as for two control pigs. Whereas the psoriasis score was statistically similar between the controls and TG-457, a highly significant increase in the score was seen in all piglets containing multiple copies of the transgene cassette. Furthermore, the dermatitis score was on par among all animals, implying that TG-S1 to TG-S5 displayed a phenotype reminiscent of psoriasis and not of dermatitis. Of note, lesional psoriatic skin will not necessarily engender the highest possible score, meaning that not all criteria will have to be met in order to qualify as psoriasis (Vinter et al., 2015).

In summary, we have generated Göttingen minipigs transgenic for human integrins α2 and β1. Efficacious ectopic expression in suprabasal keratinocytes in pigs with >50 copies of the transgene cassette was found to induce a severe skin abnormality. Most importantly, histological assessment of the skin did not suggest dermatitis, but rather a skin disease reminiscent of psoriasis. Although the animals died shortly after delivery, possibly because of extremely high levels or potential off-target expression of the heterologous integrins, they were alive at birth and fully matured, as judged by the autopsies. We believe that animals with an intermediate number of transgenes would likely be viable and still present with a psoriatic phenotype. To this end, transgenes could be removed from preserved transgenic porcine fibroblasts by applying CRISPR/Cas9 nucleases or excision-only transposases. Alternatively, new donor cells could be generated or, perhaps more elegantly, chimeric animals featuring a reduced number of transgenes could be generated by blastocyst complementation (Nagashima et al., 2004; Matsunari et al., 2013). Our work demonstrates that deregulated keratinocytes can instigate psoriasis in human-like tissue, and we believe that such models can contribute to a better understanding of psoriasis etiology. Previous appraisals of skin-penetrating properties of candidate drugs revealed that pig skin is highly comparable to human skin as opposed to rat, mouse and artificial skin equivalents (Schmook et al., 2001). Thus, pig models of skin inflammation should be valuable tools for development and refinement of topical therapies.

## MATERIALS AND METHODS

### Ethics statement

All animal procedures were performed in accordance with relevant guidelines and regulations and approved by the Danish Animal Experiments Inspectorate (license no. 2006/561-1230).

### Plasmids and vector construction

SB transposon-based vector constructs were generated based on the plasmid vector pT2/CMV-hITGA2.SV40-neo and pT2/CMV-hITGB1.SV40-neo, which have been previously described (Staunstrup et al., 2012). A cassette encompassing human *ITGA2* and 2AP was PCR-amplified from pT2/CMV-hITGA2.SV40-neo using a reverse primer containing the 2AP sequence. Subsequently, *ITGA2* was released from pT2/CMV-hITGA2.SV40-neo by *Bgl*II and *Cla*I digestion and substituted with ITGA2-2AP. PCR amplification of *ITGB1* was undertaken with pT2/CMV-hITGB1.SV40-neo, subsequently the amplicon was inserted in *Cla*I-digested pT2/CMV-hITGA2-2AP.SV40-neo producing the pT2/CMV-hITGA2-2AP-hITGB1.SV40-neo construct. The CMV promoter was released by *Asc*I digestion and replaced with the INV promoter and the first intron amplified from pH-3700-pL2 (kindly provided by Rikke Christensen, Dept of Biomedicine, Aarhus University, Denmark), generating pT2/INV-hITGA2-2AP-hITGB1-bGHpA.SV40-neo. pCMV-SB100X has been previously described (Mátés et al., 2009).

### Transposition and generation of donor cells for HMC

For stable transfection of NIH3T3 with pT2/INV-hITGA2-2AP-hITGB1-bGHpA.SV40-neo or pT2/CMV-hITGA2-2AP-hITGB1-bGHpA.SV40-neo, cells were seeded in 6-well plates at a density of 2×10^5^ cells/well. On the following day, the cells were transfected using 0.3 µg SB100X transposase plasmid with 1.2 µg transposon vector mixed with Fugene 6 (Roche, Basel, Switzerland) according to the manufacturer's protocol using a 3 µl:2 µg ratio. After 24 h, the cells were trypsinized and transferred to a P10 dish (Greiner Bio-one GmbH, Frickenhause, Germany). After a further 24 h, the cells underwent G418 (Invitrogen) selection (1 mg/ml) for 14 days. Subsequently, the resistant colonies were isolated and pooled for RNA or protein extraction. Primary male Göttingen fibroblasts were seeded, transfected and selected as described above, using 20 ng SB100X plasmid and 1 µg transposon vector along with Fugene 6 at a 3 µl:2 µg ratio. Pooled transgenic cells were re-seeded in one well of a 6-well dish and allowed to expand until confluency. At confluency, the cells were re-seeded into 4 wells of a 12-well dish at a density of 3×10^4^ cells/well and allowed to expand for 3 days to reach confluency without change of medium, ensuring G1 phase synchronization.

### Handmade cloning (HMC) and transfers

HMC was performed as described before (Du et al., 2007; Schmidt et al., 2010). Briefly, cumulus cells were removed from matured cumulus-oocyte complexes (COCs) with 1 mg/ml hyaluronidase. After partial digestion of zona pellucida with 3.3 mg/ml pronase, oriented bisection of oocytes was performed to remove nuclei. Each cytoplast (without polar body) was attached to a single transgenic fibroblast and fused in fusion medium (0.3 M mannitol, 0.1 mM MgSO_4_ and 0.1 mg/ml PVA) in a fusion chamber (BTX microslide 0.5 mm fusion chamber, model 450; BTX, San Diego, CA, USA) with a single direct current (DC) impulse of 2.0 kV/cm for 9 μs. One hour later, each cytoplast-somatic cell pair was fused with another cytoplast in activation medium (fusion medium with 0.1 mM CaCl_2_) by a single DC pulse of 0.86 kV/cm for 80 μs. After incubation in porcine zygote medium 3 (PZM-3) supplemented with 5 µg/ml cytochalasin B, 10 µg/ml cycloheximide for 4 h, the reconstructed embryos were cultured in PZM-3 medium for another 5-6 days to develop into transgenic blastocysts. Blastocysts and morulae, at day 5 and 6, were surgically transferred into recipient sows (Schmidt et al., 2010).

### Cells

The murine fibroblast cell line NIH3T3 and human keratinocyte cell line HaCaT were cultured in Dulbecco's modified Eagle's medium, DMEM (Lonza, Verviers, Belgium) with 10% fetal calf serum (FCS; Lonza, Verviers, Belgium). Established Göttingen primary fibroblasts were maintained in DMEM with 15% FCS. All media were supplemented with 100 U/ml penicillin, 0.1 mg/ml streptomycin and 265 mg/l L-glutamine and all cells were maintained at 37°C, 5% CO_2_. Outgrowth and expansion of primary fibroblasts and keratinocytes was achieved from explants derived from pig ear biopsies. The epidermis was sectionally isolated and the explants were placed in 25 cm^2^ culture flasks (TPP, Trasadingen, Switzerland) and incubated bottom-up overnight at 37°C, 5% CO_2_. Fibroblast outgrowth was promoted in AmnioMAX-C100 (Gibco, Invitrogen, Paisley, UK). Outgrowth and expansion of keratinocytes was achieved in 15% FCS DMEM containing the additives as described above plus 10 ng/ml EGF (Gibco), 50 µM gentamycin and 0.4 µg/ml hydrocortisone (Sigma-Aldrich) at 37°C, 5% CO_2_ for 7-10 days, allowing keratinocytes to migrate from the explants. Thereafter, the medium was substituted with epidermal growth factor (EGF) and bovine pituitary extract (BPE) containing serum-free keratinocyte medium, K-SFM (Gibco, Invitrogen, Paisley, UK). Outgrowth of melanocytes and fibroblasts is selected against as a result of the low calcium concentration of the medium. Authentication of cell type and monitoring of cell culture contamination was performed by visual inspection.

### Western blot analysis

NIH3T3 cell lysates were prepared in lysis buffer 9803 (Cell Signaling Technology) containing a cocktail of protease inhibitors (Roche, Basel, Switzerland). Keratinocyte cell lysates were prepared in a lysis buffer containing 50 mM Tris-HCl pH 7.6, 2.5% SDS, 10% glycerol, 10 mM β-glycerophosphate, 10 mM NaF, 95 µM sodium orthovanadate and a cocktail of protease inhibitors (Roche). Total protein content was determined using a BCA Protein Assay Kit (Pierce). Lysate corresponding to 10 µg total protein was subjected to PAGE using 3-8% gradient gels (Bio-Rad). Proteins were transferred to nitrocellulose membranes using the iBlot transfer system (Invitrogen) at 12 V for 25 min. Human β1 integrin was detected using anti-human β1 integrin rabbit polyclonal antibody (Millipore, AB1952) at a 1:1000 dilution or mouse anti-human β1 integrin monoclonal antibody (BD transduction laboratories, 610467) at a final concentration of 0.1 µg/ml. Human α2 integrin was detected using anti-human α2 integrin mouse monoclonal antibody (BD transduction laboratories, BD611016) at a 1:1000 dilution. Actin was detected using a monoclonal anti-actin antibody, (Sigma-Aldrich, A4700) at 1:2000. Secondary antibodies coupled to HRP (DAKO) and ECL Western Blotting Detection Reagents (GE Healthcare) were used for visualization.

### PCR and RT-qPCR

PCR was performed on 100 ng gDNA extracted from primary fibroblasts originating from the transgenic pigs or non-transgenic pig controls. Primers specific for the neomycin selection marker (500 bp product), *ITGB1* (280 bp product) and *ITGA2* (175 bp product) transgenes, and for the SB100X transposase (390 bp product) were used in a standard Taq polymerase (Ampliqon, Skovlunde, Denmark) PCR assay. Reactions were run for 25 cycles and visualized on a 1.5% agarose ethidium bromide stained gel. For qRT-PCR, 150-300 ng total RNA (RNeasy kit, Qiagen, Hilden, Germany) from transfected NIH3T3, Göttingen primary keratinocytes and from skin biopsies was DNase treated (DNA-free, Ambion, TX, USA) and used for standard 20 µl cDNA synthesis (AffinityScript cDNA synthesis kit, Stratagene). Of total cDNA, 1:20 to 1:10 was used per qPCR reaction. Q-PCR analyses were performed according to the manufacturer's instructions with a LightCycler 400 (Roche). The following TaqMan gene expression assays (Applied Biosystems) were deployed; human ITGA2 detection (Hs00158127), human ITGB1 (Hs00559595).

### Southern blot analysis

Between 11 and 15 µg of column-purified genomic DNA was digested overnight at 37°C in a total volume of 200 µl with the following restriction enzyme combinations; *Eco*RV+*Pvu*II or *Eco*RV+*Sal*I (New England Biolabs). The digested DNA was precipitated with sodium acetate and resuspended in 20 µl TE. The DNA fragments were resolved on a 0.8% agarose gel overnight at 1 V/cm and transferred to a nylon membrane (Amersham Hybond-N^+^, GE Healthcare) by vacuum suction. During transfer, the DNA was nicked, denatured and neutralized in 0.25 M HCl, 1.5 M NaCl+0.5 M NaOH and 1.5 M NaCl+1 M Tris-HCl pH 7.6, respectively. Subsequently the membrane was washed with 20×SSC. The DNA was UV-crosslinked to the membrane and pre-hybridized with 0.4 mg/ml salmon sperm ssDNA (D1626, Sigma-Aldrich) in 5×SSPE (0.75 M NaCl, 0.05 M NaH_2_PO_4_, 0.005 M EDTA), 5×Denhart's (0.1% Ficoll 400, 0.1% polyvinylpyrrolidone, 0.1% BSA), 1% SDS, 50% formamide, 5% dextran-sulfate for 4 h at 42°C. A neomycin probe of 790 bp was CTP ^32^P-labeled (Prime-It II random primer labeling kit, Stratagene) and mixed with salmon sperm ssDNA (1 mg/ml) and hybridization buffer and finally incubated with the membrane at 42°C overnight. The membrane was washed in 2×SSPE for 2×5 min at 26°C and in 2×SSPE/0.5% SDS for 2×15 min at 53°C and finally in 0.2×SSPE/0.5% SDS for 1×5-15 min at 53°C. The membrane was air-dried and enclosed in a cassette for exposure of an X-ray film (Konica Minolta Medical and Graphic).

### Immunocytochemistry

Skin explants from α2β1 transgenic and non-transgenic pigs were placed in slideflasks from which primary keratinocytes were expanded. After 2 weeks, the explants were removed and the cells fixed in 4% formalin for 5 min, washed in TBS+0.05% Tween 20 (TBS-T) and blocked in 1% BSA, 0.3% Triton X-100 in PBS for 30 min at ambient temperature. Subsequently, the cells were incubated overnight at 4°C with mouse monoclonal antibodies against human β1 integrin (P5D2, kindly provided by Uffe Birk Jensen, Dept of Biomedicine, Aarhus University, Denmark) at a 1:100 dilution or mouse monoclonal antibodies against human α2 integrin (Santa Cruz Biotechnology, P1E6) at a 1:50 dilution in 100 µl, 1% BSA, 0.3% Triton X-100 in PBS. The cells were washed in 0.05% TBS-T and incubated with an Alexa Fluor 488 (green) conjugated goat anti-mouse IgG secondary antibody (Invitrogen, A11029) at a 1:400 dilution in 100 µl of 1% BSA, 0.3% Triton X-100 in PBS for 1 h at room temperature and once again washed in 0.05% TBS-T. For dual staining, an Alexa Fluor 647 (red)-conjugated goat anti-mouse IgG secondary antibody, A21236 (Invitrogen, Paisley, UK) was used to label human α2 integrin. The slides were mounted in Vectashield mounting medium (Vector Laboratories) containing 1.5 µg/ml DAPI and visualized with a Leitz DMRB microscope (Leica Microsystems). Importantly, the primary antibodies were class-specific, as demonstrated by the fact that anti-human β1 antibody did not bind α2 integrin in *ITGA2*-containing keratinocytes and anti-human α2 antibody did not recognize β1 integrin in *ITGB1*-containing cells.

### H&E staining

Formalin-fixed paraffin-embedded (FFPE) blocks of skin from transgenic and non-transgenic pigs were sectioned in 4 μm slices deparaffinized in xylene and rehydrated. The skin samples were placed in 0.1% Mayer's Hematoxylin (Sigma-Aldrich) for 15 min and rinsed in copious amounts of tap water. The slides were then successively dipped in 0.5% Eosin for 2 min and in ddH_2_O until the Eosin stopped streaking. The samples were dehydrated in increasing concentrations of ethanol and lastly in xylene before being mounted with Clarion Mounting Medium (Sigma-Aldrich).

### Immunohistochemistry

Immunohistochemical staining was performed on BenchMark XT (Ventana Medical Systems, Tucson, AZ, USA) by an indirect sequential immunoenzymatic technique. Sections of 4 mm were cut from each paraffin-embedded tissue block, mounted on Superfrost Plus slides (Thermo Fisher Scientific), and dried for 5 min at 60°C. Standard settings and reagent kits of Benchmark XT (Ventana Medical Systems) were used in deparaffinization, rehydration, antigen retrieval and endogenous peroxidase blocking steps. Rabbit anti-CD3 monoclonal antibodies (ready to use, Ventana Medical Systems, 790-4341), mouse anti-CD66b monoclonal antibodies (1:600, BD Biosciences, 555723), mouse anti-CD163 monoclonal antibodies (1:450, Serotec, MCA 1853) and rabbit anti-Ki-67 monoclonal antibodies (1:200, Cell Marque, 275R-16) were incubated for 20 min (anti-CD163) or 32 min (anti-CD3, anti-CD66b and Ki-67) at room temperature followed by Ventana ultraView Universal 3,30-diaminobenzidine (DAB) Detection Kit that includes HRP-conjugated anti-mouse and anti-rabbit antibodies. Slides were counterstained with Mayer's Hematoxylin and bluing reagent and later manually dehydrated and mounted.

### Confocal microscopy

Transgenic or control pig keratinocytes were seeded at a density of 10^4^/chamber in an 8-well poly-L-lysine coated 1 µm slide plate (Ibidi, Munich, Germany). After incubation for 24 h, the cells were fixed, permeabilized and stained with mouse anti-human monoclonal antibodies P5D2 or mouse anti-human monoclonal antibodies P1E6, followed by incubation with the secondary Alexa Fluor 488 goat anti-mouse IgG Ab as described above. The cells were visualized utilizing a 488 nm line of a multiline argon laser (detection of Alexa Fluor 488) and the 405 nm line of a 405-430 nm diode laser (detection of DAPI) in a confocal laser scanning microscope (LSM 710, Zeiss) using 63× oil-immersion objective with a numerical aperture of 1.4.

### Flow cytometry

Transgenic and non-transgenic primary porcine keratinocytes expanding from skin explants were harvested by trypsinization and fixed in 4% formalin for 10 min. After washing with cold 0.5% TBS-T, cells were permeabilized and blocked in PBS containing 0.2% saponin (Sigma-Aldrich) and 0.5% BSA at 4°C, 30 min. All samples were washed in 0.5% TBS-T and stained for α2 integrin (P1E6, 1:50) or β1 integrin (P5D2, 1:100) for 4°C, 60 min. Hereafter, the cells were washed in 0.5% TBS-T and subsequently incubated with the highly cross-adsorbed Alexa Fluor 488 goat anti-mouse IgG (Invitrogen, A11029) at 1:400 and 4°C for 60 min. The cells were washed and resuspended in 300 µl PBS (no MgCl_2_, no CaCl_2_). Finally, 10,000 events were analyzed on a BD FACSAria III machine using BD FACSDiva and FlowJo software.

### Epidermal thickness

The mean epidermal thickness (epidermal area divided by epidermal length) was calculated using the Visiopharm Integrator System (VIS) (Visiopharm, Hørsholm, Denmark).

### Sensitization

A shaved area on the left dorsal flank of the pigs was sensitized with 200 µl of 10% fluoro-2,4-dinitrobenzene (DNFB; Sigma) on day 0. Following sensitization, tissues were treated three times a week with 200 µl of 1% DNFB until day 43. Five-mm punch biopsies were collected from untreated skin at day 0 and from the sensitized area on day 37, 44, 51, and, in the first round of experiments, on day 58. One half of the biopsy (cut longitudinally) was kept in 4% formalin and the other half was kept in 1 ml RNAlater (Sigma). Gene expression analyses were performed on skin biopsies from the area on the back exposed to DNFB. RNA was purified using Qiagen RNeasy Lipid Tissue Mini Kit. cDNA was synthesized using an Applied Biosystems High-Capacity cDNA Reverse Transcription Kit. Gene expression analyses were performed using pre-designed Applied Biosystems TaqMan assays according to the manufacturer's instructions; porcine CXCL10 (Ss03391846), porcine PCNA (Ss03377029), porcine STAT1 (Ss03392291), and porcine STAT3 (Ss03388439).

### Histological score

H&E-stained tissue sections were blinded and evaluated by two observers, of which one was a trained dermatopathologist, to compare the histological features of psoriasis and dermatitis. The following characteristic psoriatic parameters were assessed: parakeratosis, neutrophil infiltration in epidermis, increased epidermal thickness (acanthosis), absence of stratum granulosum, psoriasis-like papillary pattern (rete ridges), thinning of the epidermis over the dermal papillae, dilatation of vessels in the dermal papillae and presence of neutrophils outside the vessels in the dermal papillae. The following characteristic dermatitis parameters were assessed: orthokeratosis, retained stratum granulosum, acanthosis, spongiosis, exocytosis and occurrence of eosinophil leukocytes. The histological score for each pattern was graded on a scale from 0 to 2 (0, absent; 1, present; 2, severe) (Nagashima et al., 2004).

### Statistics

*P*-values were calculated by a two-tailed Student's *t*-test or by Fisher's exact test, where appropriate, to test the null hypothesis of no difference between the compared groups. The assumption of equal variances was tested by the *F*-test. In all statistical analyses, *P*<0.05 was considered significant.
